# Supplementary material for: Evaluation of Cardiovascular Toxicity of Folic Acid and 6S-5-Methyltetrahydrofolate-Calcium in Early Embryonic Development
Source: Cells. 2022 Dec 7;11(24):3946. doi: 10.3390/cells11243946 (PMC9777352; doi:10.3390/cells11243946)
Supplement: Supplementary file 1 [file cells-11-03946-s001.zip › cells-2013795-supplementary.pdf]

Supplemental Information

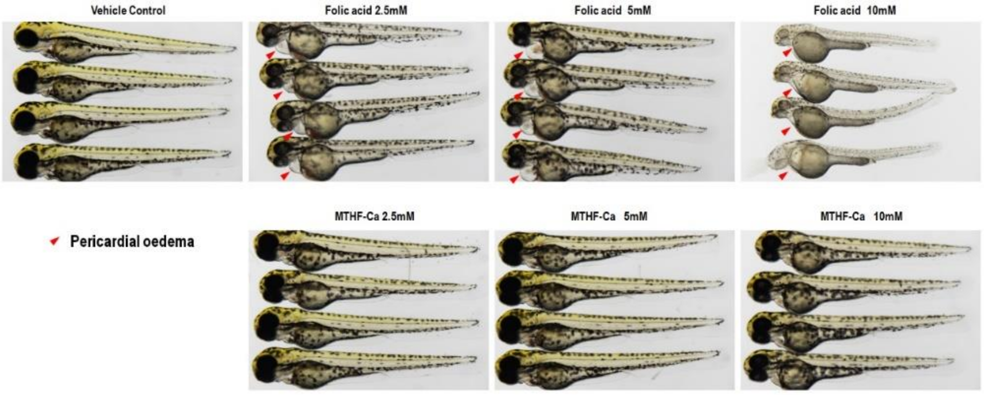

Figure S1. FA, but not MTHF-Ca, causes cardiotoxicity in zebrafish. Representative bright-field images of zebrafish embryos at 3 days postfertilization (dpf) treated with vehicle control (fish water), FA (2.5, 5, and 10 mM), or MTHF-Ca (2.5, 5, and 10 mM). Compared with control or MTHF-Ca, FA-treated embryos present pericardial oedema and reduced contractile force (*n* = 40 each group). FA: folic acid; MTHF-Ca: 6S-5-methyltetrahydrofolate-calcium.

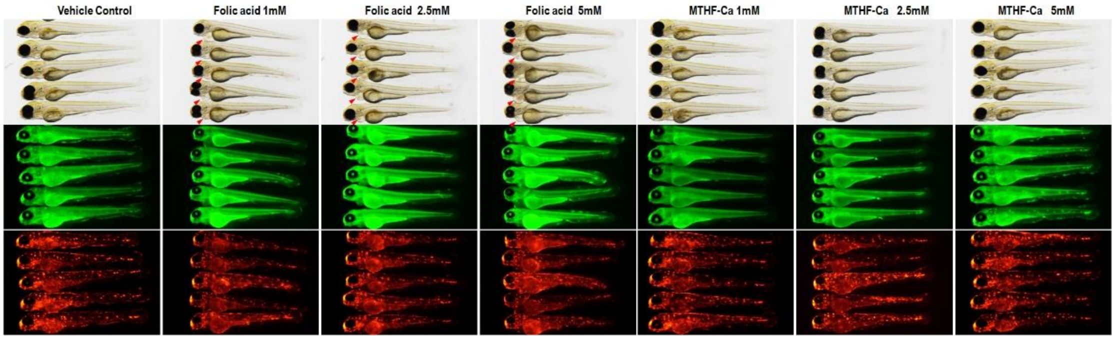

Figure S2. FA and MTHF-Ca do not induce heart-specific apoptosis in zebrafish. There is no heart-specific apoptosis induced by either FA or MTHF (*n* = 40 each group). FA: folic acid; MTHF-Ca: 6S-5-methyltetrahydrofolate-calcium.

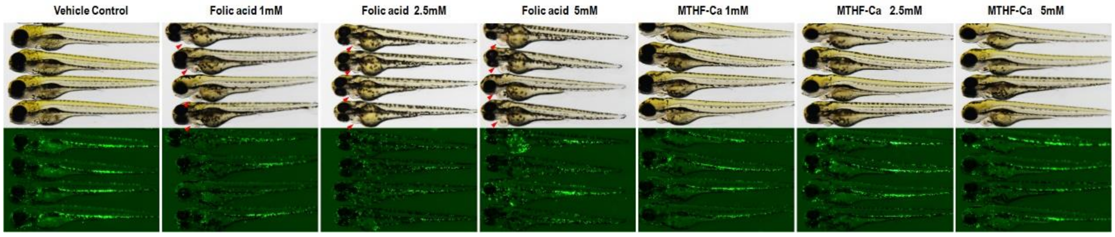

Figure S3. FA and MTHF-Ca do not induce heart-specific macrophage migration. There is no heart-specific macrophage migration induced by either FA or MTHF (*n* = 40 each group). FA: folic acid; MTHF-Ca: 6S-5-methyltetrahydrofolate-calcium.

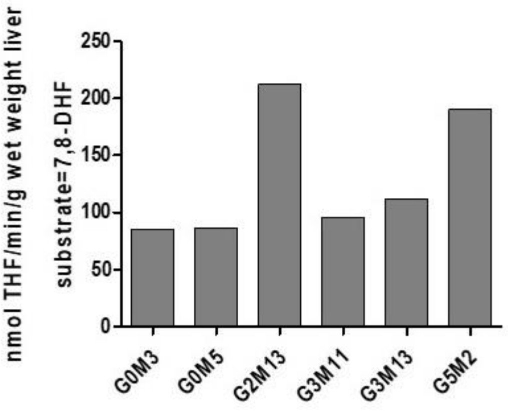

Figure S4. DHFR activity in livers of mice is high. A previous study demonstrated that dihydrofolate reductase (DHFR) activity in rat livers is much higher compared to that in humans [**8**]. In the current study, we found that DHFR activity in the livers of mice was high (130.21 ± 22.97 nmol/THF/min/g wet weight liver). Livers of female mice were frozen in liquid nitrogen and stored at -80 °C. Prior to the analysis, the liver tissues were homogenized on ice in a homogenizer (Jingxin, Shanghai, China) with 1 mg liver: 20 µL homogenizing buffer (C3228, Sigma-Aldrich, Louis, USA) containing a protease-inhibitor cocktail. The homogenate was centrifuged at 100,000 g for 30 min at 4 °C, and the supernatant was removed for DHFR-activity assay. The activity was monitored using a commercial dihydrofolate reductase assay kit according to manufacturer's specifications (CS0340, Sigma-Aldrich, Louis, USA) and read on the microplate reader at 340 nm. Among six female mice, the levels of DHFR activity in the liver, with 7,8-DHF as substrate, varied from 70 to 220 nmol THF/min/g, but was higher than that in humans.

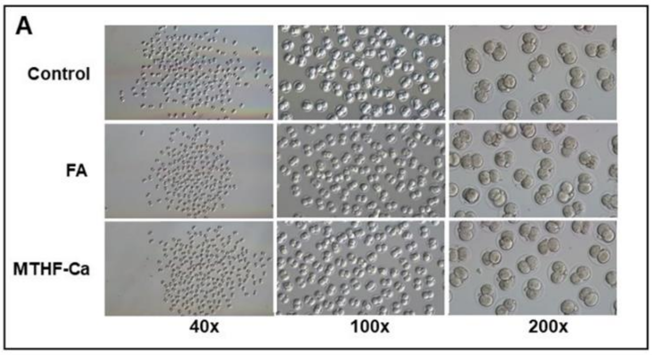

B

	24h . Number of fertilizations	Number of unfertilized	48h Number of fertilizations	Number of unfertilized	72h Number of fertilizations	Number of unfertilized
**Control**	800 (84.21%)	150 (15.79%)	455 (15.79%)	345 (43.13%)	217 (85.10%)	38 (14.90%)
**FA**	832 (81.17%)	193 (18.83%)	421 (50.60%)	411 (49.40%)	188 (85.10%)	33 (14.93%)
**MTHF-Ca**	900 (87.04%)	134 (12.69%)	420 (46.67%)	480 (53.33%)	200 (90.90%)	20 (9.09%)

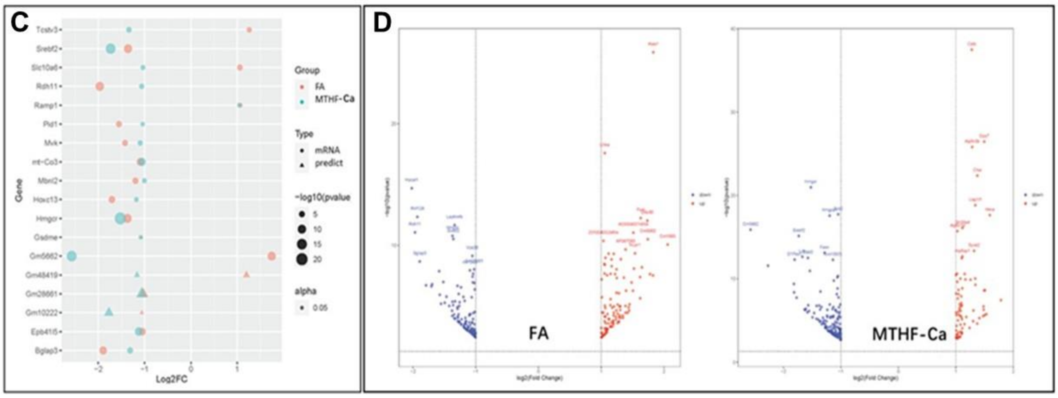

Figure S5. Images of mouse blastocysts at 24 h, 48 h, and 72 h, cultured with FA and MTHF-Ca treatment. **(A)** Demonstration of the in vitro fertilized blastocysts of mice cultured with FA and MTHF-Ca for 24, 48 and 72 h. (**B**) Representation of the number of fertilized and unfertilized blastocysts. FA: folic acid; MTHF-Ca: 6S-5-methyltetrahydrofolate-calcium. (**C**,**D**) To explore the possible molecular mechanisms, mouse blastocysts cultured with FA and MTHF RNA-seq transcriptome analyses (n=10 each group). (**C**,**D**) Volcano maps of GDE in mouse blastocysts regulated with FA and MTHF. *Gm5662, Slc10a6*, and *Tcstv3* were upregulated by FA but downregulated by MTHF.

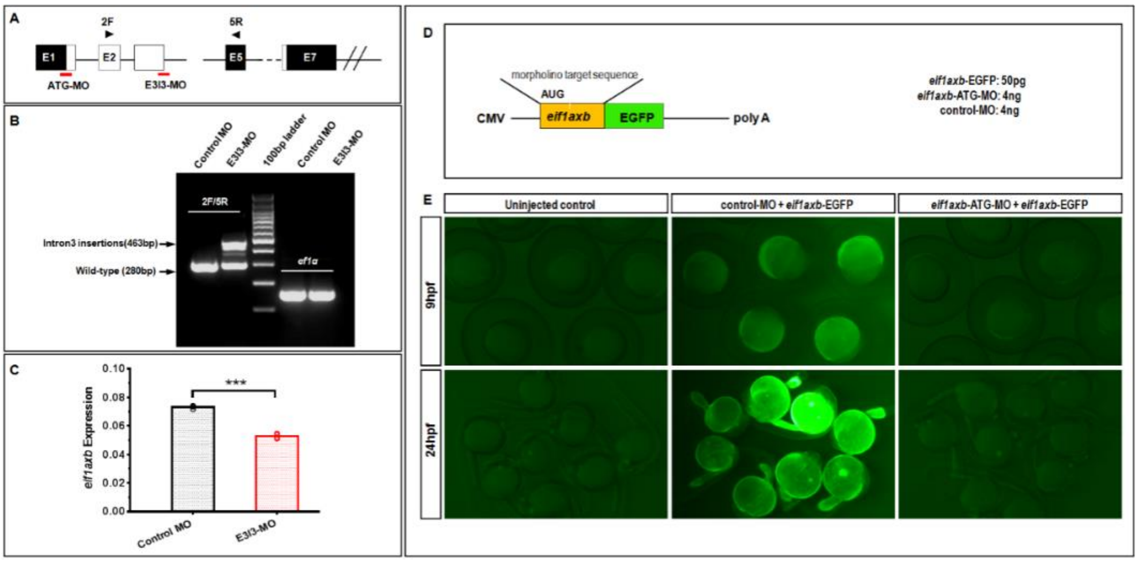

Figure S6. Effectiveness of *eif1axb* knockdown. (**A**) The zebrafish *Eif1axb* gene was targeted by specific morpholino antisense to prevent proper splicing of exon 3 (E3I3-MO). Primers 2F and 5R interrogate the presence of wild-type (nonmutant) transcripts or those in which intron 3 has been inserted. (**B**) RT-PCR transcript of *Eif1axb* from control-MO and E3I3-MO morpholino-injected embryos 1 day after fertilization, demonstrating insertion of intron 3. Injection of 4 ng *Eif1axb* morpholino alters the splicing between exon 3 and intron 3, as revealed by the shift in PCR bands between control and *Eif1axb* morpholino-injected embryos. (**C**) Quantitative measurements of *Eif1axb* expression levels measured by qRT-PCR (***$P < 0.0001$). MO-targeted downregulation of *Eif1axb*. Samples were collected 1 day postfertilization after introduction of 4 ng MO at the one-cell stage ($n = 20$). (**D**) Schematic diagrams of *Eif1axb*-EGFP fluorescent reporter mRNAs, the upper one containing the *Eif1axb*-ATG-MO target sequence (yellow box) fused in-frame with EGFP. (**E**) 50 pg *Eif1axb*-EGFP injected with a standard control morpholino (4 ng) or *Eif1axb*-ATG-MO (4 ng). Embryos were photographed at 9 and 24 hours postfertilization. Embryos injected with *Eif1axb*-GFP plasmid DNA under the driving of CMV promoter showed green fluorescence. When co-injected with *Eif1axb*-ATG-MO, green fluorescence decreased dramatically.
